# Toward Measuring Chinese EFL Teachers’ Resilience: The Role of Teachers’ Enjoyment, Anger, and Anxiety

**DOI:** 10.3389/fpsyg.2022.853201

**Published:** 2022-06-03

**Authors:** Lu Gan, Yonggang Gao, Jinwen Wu

**Affiliations:** School of Foreign Languages, China University of Geosciences Wuhan, Wuhan, China

**Keywords:** Chinese EFL teachers, anger, anxiety, enjoyment, resilience

## Abstract

Teachers have been regarded for many years as one of the most impactful elements with a significant function in educational and learning contexts. Several studies have been conducted on teachers and their performances in the classes. Positive psychology has focused on both the constructive and deconstructive feelings that teachers encounter in the process of teaching. Among the investigated elements, enjoyment anger, and anxiety can be regarded more significant in the relevant literature. The current research, thus, clarifies their association and connection with Chinese language educators’ resilience. To this end, 464 male and female Chinese EFL teachers participated in the present study, and their enjoyment, anger, anxiety, and resilience were inspected by completing the associated questionnaires. The data analysis indicated that there is a substantial correlation between teachers’ resilience and their emotions. Similarly, it is concluded that the best predictor of teachers’ resilience is enjoyment. Also, some academic suggestions for the study regarding the development of teachers’ resilience in educational situations are proposed.

## Introduction

The instructing profession is one of variation and challenge due to its cognitive, emotional, and serviceability quality. Educators must constantly be prepared for different variations and growths in the setting, specifically the scholastic one (Hasbay and Altindag, 2018; Mercer, 2020). Nonetheless, educators who abandon variation will 1 day be banished and replaced by those who can cope with variation well (Fadli and Rukiyati, 2020). Educators can encounter a variety of mental dispositions that can influence classroom practices and conducts, such as self-efficacy, loss, and stress (Pishghadam et al., 2014). Coping with stress varies from person to person, and those who cannot deal with it will have burnout at work so being resilient is one of the coping techniques that educators can carry out (Diasti, 2021). Resilience is a novel model in psychosomatic research that formerly emphasized finding the constructive aspects of emotions to predict the deconstructive aspects that allow educators to bounce back and forth from destructive stressors and distressing procedures in the field (Fathi and Saeedian, 2020).

Alternatively, there has been significant growth in the attention paid to the role that emotion plays in language education recently (Bigelow, 2019). Prior (2019) mentioned that feelings have never been entirely disregarded, but motivated scholars are accustomed to them “creatively and critically.” Studies on emotions in Second Language Acquisition (SLA) are thriving and growing and this is mirrored by the growing numbers of specific issues and edited volumes on this subject (Dewaele and Li, 2020). Regardless of the latest studies emphasize the teachers’ emotions (Cowie, 2011; King, 2016), the study of emotions in second or foreign language education had been disregarded for a long time because of the field’s cognitivist underpinning and it is about time to solve this emotional deficiency (Dörnyei and Ryan, 2015; Martínez Agudo, 2018). In foreign language education, the interest in feelings preceded the rise of Positive Psychology (PP) in this area that upholds a more comprehensive standpoint about people (Dewaele and MacIntyre, 2016; Wang et al., 2021), and SLA scholars have newly focused on constructive feelings in foreign language students, after the emergence of PP.

Studies on educators and education over the last 20 years have repeatedly shown that education is an emotionally, physically, and cognitively challenging task (Wassell and Lavan, 2009; Zembylas and Schutz, 2009). Cowie (2011) mentioned that teaching English is a very emotional task so it is important to investigate elements that are related to or might have some influence on the development of educators’ feelings, following the confirmed strong role that educators’ feelings play in the education and learning cycles (Becker et al., 2014). Educators in the classroom experience a range of emotions that affect their performance and learners’ performance. Frenzel (2014) distinguished seven individual emotions educators experience in class, namely, joy, pride, rage, anxiety, humiliation, boredom, and compassion and these emotions are directly associated with other emotional-inspirational concepts that educators may experience in class. Furthermore, language education is a cycle full of deconstructive and constructive emotions; thus, developing a constructive educational setting must be the first goal of a language educator (Méndez and Peña, 2013). Dewaele and MacIntyre (2016) believe that both constructive and deconstructive feelings have adjective functions, which cooperate in advancing foreign language education, and more powerful overall emotional encounters establish the inspiration for foreign language education.

Second language education is denoted as moving away from focusing solely on deconstructive feelings such as foreign language classroom anxiety (FLCA), and anger and moving toward considering students’ constructive feelings such as foreign language enjoyment (FLE; Dewaele and MacIntyre, 2014; Dewaele and Li, 2020). Hagenauer et al. (2015) found that educators’ feelings such as joy, anger, and worry are formed through relational connections with learners, discipline in class, and learners’ involvement in class activities. For instance, anger emerges as the leading deconstructive feeling for educators, as reported in qualitative and narrative studies of educators’ feelings (Frenzel, 2014). However, these present studies show that educators’ emotions are a relevant result and are associated with general educator wellbeing, risk of burnout, and persistence in the instructing profession (Chang, 2013). Moreover, since educators’ sensations are associated with the quality of instruction and the established connections with the learners, they are suggested as being pertinent to learners (Sutton and Wheatley, 2003; Klassen et al., 2012). The multi-factor conceptualization established by Scherer (2000) was utilized in characterizing educators’ emotions and this multi-factor conceptualization of emotions is a Teacher Emotions Scales (TES) method that is sensitive to emotional encounters but differs significantly in terms of individual phenomenology and associated inspirational inclinations and nonoral practices. Reviews of the qualitative studies in the educational setting, in specific, demonstrated that enjoyment can be regarded as the most important constructive feeling (Sutton and Wheatley, 2003; Frenzel, 2014).

Enjoyment is considered as a noble emotional state extending away from one to accomplish something different or even unexpected; principally in copying with some stimulating and demanding activities that improve particular development and persistent wellbeing (Csikszentmihalyi, 2014). Foreign language enjoyment is explained as an intricate feeling, catching the associating aspect of the challenge and discerned capability that mirrors the human drive for achievement when encountering hard tasks (Dewaele and MacIntyre, 2016). Enjoyment is a strong incentive for SLA (Dewaele and Alfawzan, 2018; Pavelescu and Petri’c, 2018; Piniel and Albert, 2018) and it is a particularly interesting constructive feeling that refers to the feeling people experience when they go beyond just meeting their demands to achieve something unprecedented or amazing (Csikszentmihalyi, 2014).

Contrastingly, anger is one of these possibly mismatched emotions. It is one of the most extreme and possibly risky emotions because it is connected with a powerful inclination to act (Harmon-Jones and Harmon-Jones, 2016). Thus, anger appears to be the most important deconstructive feeling for educators as is reported in qualitative and narrative studies on educators’ feelings (Chang, 2013; Frenzel, 2014). Aside from enjoyment and anger, we incorporated anxiety due to having attained a lot of research interest overall, but also in the educational setting and it is the most explored feeling, among deconstructive ones, in both instructional and foreign language studies (Dewaele and MacIntyre, 2014).

Indeed, there is considerable research on students’ emotions (Dewaele and MacIntyre, 2014; MacIntyre et al., 2019), but relatively little research focuses on educators’ encounters with specific, distinct emotions like enjoyment, anger, and anxiety (Frenzel, 2014; Keller et al., 2014). In addition, inquiries have displayed that during class time, educators experience diverse emotions, like enjoyment (Sutton and Wheatley, 2003; Frenzel et al., 2009), anger (Kuppens et al., 2008; Chang, 2013), and anxiety (Beilock et al., 2010; Keller et al., 2014). Indeed, educators who echo more positive emotions (enjoyment) tend to have minor burnout and greater career fulfillment (Brackett et al., 2010) and likewise, educators’ constructive emotions cultivate and nurture learners’ success (Fredrickson, 2003; Frenzel et al., 2009). Based on the above-mentioned studies and their effect on language learners, to the best of the researchers’ knowledge, not enough studies so far have been carried out on the role of these types of emotions, namely, enjoyment (Guo, 2021), anger, and anxiety on teachers’ resilience (Xue, 2021; Wang et al., 2022) in the Chinese EFL context. To this end, the following study is going to answer the following questions:

*Q1.* Is there any significant relationship between EFL teachers’ resilience and their emotions?

*Q2.* What component of teachers’ emotions can best predict their resilience?

## Review of the Literature

### Emotion

As stated by Oatley (2000), the term feeling has been used in various forms to explain different abstract points of view, namely, physiological, hypothetical, sociological, organizational, hominoid, and spiritual ones. Numerous theorists conceptualize feeling as a multi-faceted process; however, there is no consensus between character and societal psychologists about what embraces feelings (Frijda, 2001). For instance, Sutton and Wheatley (2003) examined emotional factors such as assessment, individual experience, physiological alterations, emotional expressions, and behavioral inclinations. One more example is Zembylas (2004), who identified emotions as conceptual, evaluative, and political, which are shaped by politics and the balance of power in schools and society. Recently, Izard (2010) argued that while there seems to be some consensus on the form and role of emotions, the definition of emotions remains suggestive. Currently, there is a consensus that emotions are made up of several factors. In other words, every emotion contains a large collection of more or less chaotic factors that are prompted together by event evaluation and factor propensities.

Emotion is characterized as socially developed, individually performed ways of being that arise from the cognizant and/or insensible perceptions about discerned achievements at reaching objectives or sustaining principles or convictions in communications as part of socio-historical circumstances (Schutz and Lee, 2014). Moreover, while there seems to be some consensus on the form and role of feelings, the meaning of feeling remains suggestive (Izard, 2010). Currently, there is a consensus that feeling is made up of several elements. In other words, every feeling contains a large compilation of rather chaotic elements that are stimulated together by how an occurrence is evaluated as well as by element bias (Scherer, 2000). Fried et al. (2015) examined the review on educator emotions and discovered that the connection between educator emotions and other important elements produces noteworthy outcomes. The outcomes of these studies highlight the importance of educators’ emotions in class and their potential impact on class life. It has also been demonstrated that educator emotions are closely related to learners’ emotions (Meyer and Turner, 2006). The educator trainers reported that there was a constructive connection between educator and learner joy in the classroom (Frenzel et al., 2009). Newberry (2010) maintained that educators needed guidance or support in building personal connections with their learners. This is because it involves considerable emotional work regularly. Classrooms grouped according to constructive emotions toward education and learning appear to offer optimal conditions for learners’ growth and performance (Yan et al., 2011).

### Resilience

In the field of psychoanalysis and developing psychology, the concept of resilience emerged due to a flourishing focus on individual attributes or features that allow some kids to adjust constructively and endeavor despite many challenges (Waller, 2001). The capability of recovering or arising from a deconstructive emotional encounter is known as resilience (Mansfield et al., 2016). A cycle of adjustment in which educators use techniques to solve the difficulties they encounter in their setting is known as resilience (Mansfield et al., 2016; Ainsworth and Oldfield, 2019). This characterization of resilience is significant to this research as it suggests that resilience growth is a “cycle” that educators “adjust to” solve difficulties and this adjustment is made in their setting through their practices (Ainsworth and Oldfield, 2019; Toktas, 2019). Educator resilience is characterized as the ability to recover and regain ones’ strengths and spirit rapidly and efficiently despite difficulty and is thoroughly related to strong purpose, self-efficacy, and inspiration to instruct (Gu and Day, 2007). Another significant resource for educators trying to comprehend and build wellbeing in their learners in hard circumstances is working on positive psychology. There has been a specific emphasis on developing students’ resilience, reflecting growing worries in conventional general teaching (Seligman, 2011). It has also been contended that educator resilience is a “quality retention” problem with the retention of devoted, interested, and inspired educators who, despite the work phase, keep on growing professionally and optimizing their competency to offer high-quality education (Gu and Day, 2007). Resilience alludes to the cycle of survival post encountering a demanding circumstance incorporating dynamic frameworks that effectively adjust to disturbances that jeopardize the functioning, practicality, or growth of individual frameworks and the cycle of change toward a new static functional condition (Masten, 2016; Guo, 2021). The accumulation of a person’s capabilities that allow him/her to recover from a challenge and even endeavor during hard times is known as resilience (Kim and Kim, 2017).

### Enjoyment

Boudreau et al. (2018) differentiated enjoyment from the more basic understanding of happiness. If happiness arises solely from the execution of an activity or action, enjoyment includes additional aspects like intellectual emphasis, increased attention, and optimal challenges. Educators’ emotions, like enjoyment when instructing, have also been demonstrated to correlate with the clarity and diversity of teaching, as well as the care and support for learners (Frenzel et al., 2016). They deduced that recognition of the educator’s role in creating circumstances that facilitate learners’ education and adaptation is likely to increase educators’ enjoyment. Educators’ enjoyment has been differentiated from educators’ eagerness in that the former was considered internal and personal, and the latter was considered behavioral and perceivable; however, the two concepts have a positive correlation (Frenzel et al., 2009). Enjoyment commonly stems from the learners’ excellent performance in the class, as Frenzel (2014) mentioned that educators’ emotional antecedent are their anticipations for learners’ practice. Moreover, educator enjoyment lessens educators’ deconstructive emotions and keeps them safe from emotional fatigue (Taxer et al., 2019).

### Anxiety

The concept of anxiety is multi-layered because individuals who encounter FLCA tend to feel anxious when taking part in language education and/or use (Horwitz, 2017). Anxiety is explained as a disturbing fear, or as undirected wakefulness after recognizing a threat, and as a condition of discomfort and concern, specifically about future worries (Yükselir and Harputlu, 2014). More broadly, language anxiety is affected by inner mental cycles, intellectual and emotional conditions, among other things, along with contextual demands and the presence of others, as seen on various time scales (MacIntyre, 2017). As stated by Frenzel (2014), educators are reported to be less anxious than learners because they do not face obvious setbacks as often as learners. Nonetheless, anxiety appears to be more prevalent in trainee educators because of the intricacy of learning to instruct and the ambiguity of attaining objectives while associating with guardians (Frenzel, 2014). Nevertheless, anxiety appears to be more recurrent among beginner educators because of the intricacy of studying to instruct and the ambiguity of attaining objectives, as well as when associating with guardians (Sutton and Wheatley, 2003).

An educator’s level of anxiety is influenced by numerous elements. Only some of the elements adding to the level of stress and its effects include gender, experience, type of school, physical state of the class and school, character, learners’ attributes, connection with administrators and learners’ guardians, setting, class grade level, family issues, financial issues, national or regional curriculum changes (Aslrasouli and Vahid, 2014). Shillingford-Butle et al. (2012) point out that educators face many difficulties that can cause symptoms of anxiety, such as those involving education, law, school reform policies, educator-guardian connections, and conflicts with other educators. There is a belief that the situation in which educators work forces them to perform their work badly. For instance, taking into account the physical state of language institutions, Iran almost always has old buildings with poor ventilation and shabby classrooms that bother educators.

### Anger

Anger is the most regularly deconstructive emotion stated by teachers because it can turn into anger due to many elements such as learners’ lack of discipline, difficulties, unreasonable demands, and failure to meet educators’ expectations and it is defined as a passing feeling or discomfort (Suls, 2013). For instance, educators may be angry with learners about classroom discipline and misconduct, which are the main causes of anger (Frenzel, 2014). As stated by Suls (2013), anger is an emotional element of aggression and an individual temperament associated with the feeling of being mistreated and is associated with individual arousal. Differences related to objectives like learners’ inappropriate manners or their setbacks can trigger educators’ anger (Chang, 2013). This applies specifically if educators discern their manners or setbacks as being deliberate or manageable by the learners, if they discern that learners with great capacities are having setbacks because they are not endeavoring enough or if they regard learners’ inappropriate manners (Cubukcu, 2013). Educators’ anger can also be caused by outer decreed alterations and amendments that they regard as useless to their teaching or learners. Educators who experience anger frequently are despised or subverted by others, like learners, guardians, and managers, as though their pride has been violated or their power has been jeopardized (Chang, 2013).

Based on the aforementioned points, Dewaele et al. (2019) investigated the connection between enjoyment and class anxiety and a set of educator-centric factors inside the Spanish class setting. The subjects were 210 pasts and EFL students in Spain who completed an online survey. There was a moderately deconstructive connection between enjoyment and anxiety. Subjects with an L1 English speaker as an educator were documented to have higher degrees of enjoyment and lower degrees of anxiety than those who had a foreign language speaker of English. The powerful influence of the educator on enjoyment was also demonstrated in research by Jiang and Dewaele (2019) on 564 Chinese EFL students. Enjoyment was more powerfully indicated by educator-relevant factors, while anxiety was mainly indicated by inner student factors. Qualitative analyses of students’ emotive encounters also demonstrated that anxiety was associated with the students’ conception of societal position, while enjoyment was more prone to be caused by the educator. An additional mixed methods research conducted by Dewaele et al. (2019) from 750 FL students across the globe proved that stress and joy are indicated *via* various kinds of independent factors. For one thing, joy was the most indicated by educator-centric factors like demeanor toward the educator and his/her amiability and to a less extent by students’ cultural sympathy. For another, stress was most indicated by student-internal factors like emotive balance. The analyses of periods of extreme joy and stress indicated that the educator was documented as the natural stimulant of joy while stress periods were connected to the self.

## Materials and Methods

### Participants’ Demographic Information and Data Collection Procedure

The sample comprised 464 EFL teachers with various academic qualifications containing both genders (male = 280 and female = 184) with the majority from Hubei Province (457/98%) and one municipality directly under the Central Government Beijing and other provinces (Guangdong, Henan, Jiangxi, Shaanxi, and Zhejiang; 7/2%). Their ages ranged from 18 to 58, and their teaching experiences ranged from 0 to 30 years. Their academic attainments include Bachelor degree, Master’s degree, and Ph.D. degree. Currently, they were teaching at various scholastic levels in China and were designated by using the Wechat phone app through Wen Juanxing, an online questionnaire collection tool, to collect the data. All of these were based on the respondents’ willingness. That is, before collecting the valid data, all those participants were informed of the research aim and their demographic information would be kept confidential and be used only for research purposes.

### Instruments

#### The Teacher Emotions Scales

The TES developed by Frenzel et al. (2016) contains both the general and student-group specific modified of TES and the scale is on a four-point Likert Scale ranging from strongly disagree to strongly agree. This questionnaire correspondingly made it possible to implicitly label each of the responses, which has been presented to upsurge the psychometric excellence of scales (Weng, 2004). Whereas the overall scale started with an overall beginning statement, the learner-group particular spectrums were presented with two various beginning statements based on the instructional policy that was sought in the relative context. The reliability calculated through Cronbach’s Alpha was 0.90 for the student-group detailed scales and 0.77 for the student-specific scales.

#### Connor-Davidson Resilience Scale

The scale measuring the teachers’ degree of resilience was modified from the research carried out by Connor and Davidson (2003). Although the original Connor-Davidson Resilience Scale (CD-RISC) comprises 25 items with five aspects parallel to the bases of the concept, some of the items in the original scale may not be applicable to the sample. Therefore, just 10 items were selected for the current scale parallel to the first three issues generated from the original scale, which are “the notion of personal competence, high standards, and tenacity,” “trust in one’s instincts, tolerance of negative affect, and strengthening effects of stress,” and “positive acceptance of change, and secure relationships” (Connor and Davidson, 2003). These items should be answered with a five-point Likert scale from 0 to 4 (not true at all to true nearly all the time). The internal consistency of the scale was 0.95 calculated through Cronbach α indicating satisfactory reliability.

### Data Collection Procedures

To meet the objectives of this study, by distributing the valid questionnaires online, data were collected in the middle of November 2021. Altogether, 464 valid questionnaires were gathered by the end of July and gleaned from diverse language institutes, colleges, and academies in China. To guarantee the trustworthiness of this study, all participants were fully informed of how to fill in the questionnaires and guaranteed that their answers and personal information would be remained confidential. They were also notified of their legitimacy to free withdrawal from the study at any time if they felt any discomfort. As the participants made no contact with the researcher, there were no conflicts of interest between the researcher and respondents. Then, the collected responses were double-checked for possible mistakes before being processed by SPSS software for further statistical analysis. In the final step, the probes into the research questions were conducted based on the data.

### Data Analysis

Based on the nature of the study, to answer the first research question of the study on the relationships between the variables of the study, the Pearson product–moment correlation coefficient was utilized and for the second research question, a regression was run.

## Results

The study aims to scrutinize the role of teachers’ enjoyment, anger, and anxiety on Chinese EFL teachers’ resilience. Corresponding to the research questions of the present study, Pearson’s Product–moment correlation coefficient and linear regressions were done.

*Q1.* Is there any significant relationship between EFL teachers’ resilience and their emotion?

Table 1 displays the association between teachers’ resilience and their emotions. This relationship is negative (*r* = −379), which means the greater the index of their emotions, the lower their resilience index will be. This relationship proved to be significant since *p* = 0.001.

**Table 1:** Correlations between teachers’ resilience and their emotions.

		Emotion	Resilience
Emotion	Pearson Correlation	1	−0.379
	Sig. (two-tailed)		0.001
	N	464	464
Resilience	Pearson Correlation	−0.379	1
	Sig. (two-tailed)	0.001	
	N	464	464

*Q2.* What component of teachers’ emotions can best predict their resilience?

Table 2 displays that there are significant modifications between teachers’ resilience and the components of teachers’ emotions. Considering the relationship between teachers’ resilience and enjoyment (as a positive emotion), there is a positive and significant relationship (*r* = 0.306, *p* = 0.000). The other negative emotions (anger and anxiety) had significant negative relationships with teachers’ resilience (*r* = −0.182, −0.184, *p* = 0.000, respectively).

**Table 2:** Correlations among teachers’ resilience and the components of teachers’ emotions.

		Resilience	Enjoyment	Anger	Anxiety
Pearson Correlation	Resilience	1.000	0.306	−0.182	−0.184
	Enjoyment	0.306	1.000	0.035	0.088
	Anger	−0.182	0.035	1.000	0.541
	Anxiety	−0.184	0.088	0.541	1.000
Sig. (one-tailed)	Resilience	–	0.000	0.000	0.000
	Enjoyment	0.000	–	0.224	0.029
	Anger	0.000	0.224	–	0.000
	Anxiety	0.000	0.029	0.000	–
*N*	Resilience	464	464	464	464
	Enjoyment	464	464	464	464
	Anger	464	464	464	464
	Anxiety	464	464	464	464

Table 3 tests the model fit for teachers’ resilience and their emotions. R square index showed how much of the discrepancy in the dependent variable (scores obtained from teachers’ 5emotions) was described by the model (which included resilience). In this case, the value was 0.147 articulated as a percentage; it indicates that the model (which included scores on resilience) explained 14.7 percent of the difference in scores from teachers’ emotions.

**Table 3:** Model summary for teachers’ resilience and their emotions.

Model	*R*	*R* square	Adjusted *R* square	SE of the estimate	Durbin-Watson
1	0.384^a^	0.147	0.142	3.56912	1.313

a *Predictors: (Constant), anxiety, enjoyment, and anger*.

To make sure that the model best fits the variables, Table 4 tested the hypothesis that multiple R in the population equals zero (0). The model reached statistical significance (*F* = 26.52, Sig = 0.000, this means *p* < 0.05).

**Table 4:** ANOVA for teachers’ resilience and their emotions.

Model		Sum of squares	*df*	Mean square	*F*	Sig.
1	Regression	1013.708	3	337.903	26.52	0.000^b^
	Residual	5859.773	460	12.739		
	Total	6873.481	463			

b *Predictors: (Constant), anxiety, enjoyment, and anger*.

Table 5 shows the best predictor for teachers’ resilience regarding the components of teachers’ enjoyment, anger, and anxiety. A significant regression equation was found [*F*(3, 460) = 26.52, *p* = 000]. Considering the Beta value for the components of teachers’ emotions which are 0.324, −0.112, and − 0.152, it can be concluded that the best predictor of teachers’ resilience is enjoyment.

**Table 5:** Coefficients for teachers’ resilience and components of teachers’ emotions.

S.no	Model	Unstandardized coefficients		Standardized coefficients	*t*	Sig.
		*B*	SE	Beta		
1	(Constant)	26.158	1.646		15.896	0.000
	Enjoyment	0.904	0.121	0.324	7.489	0.000
	Anger	−0.213	0.098	−0.112	−2.183	0.030
	Anxiety	−0.302	0.102	−0.152	−2.957	0.003

## Discussion

Constructive feelings, like joy, are related to resilience, adjustability, and inventiveness because they can expand a person’s idea-act collection broaden-and-build hypothesis of constructive feelings, which is specifically beneficial when encountering impediments (Fredrickson, 2003). Contrastingly, deconstructive feelings like educators’ anxiety are likely related to the inclination of educators to maintain firm authority over their instructing cycle, which can result in motivation in learners (Gloria et al., 2013; Frenzel, 2014), which could bring about demotivation in learners (Assor et al., 2005). Regarding the results of the study that enjoyment is the best predictor of resilience, it is in line with the PP theory in SLA, as the positive function of positive emotions in encouraging and facilitating language progress is assured (MacIntyre and Gregersen, 2012). Teachers experiencing a great degree of enjoyment in their classes may convey it to their learners and also cultivate not only their wellbeing and resilience but also their students’ resilience (Proietti Ergün and Dewaele, 2021). The outcomes of the study are in line with Heydarnejad et al. (2017) who concluded that teachers’ positive emotions such as enjoyment and pride were more central than destructive emotions such as anger, anxiety, embarrassment, and boredom for EFL teachers.

In addition, Jin et al. (2021) find that individuals with dispositional positivity and encountering more constructive feelings will go through better resilience. This proposes that instructing teachers on the techniques of gratefulness, constructive viewpoints, and positivity assists them with building mental assets to deal with inconveniences. Educators who documented more constructive feelings tended to be more flexible, clearer, and more understandable, connect materials to actual words more, and instruct with more excitement. In contrast, educators who encountered more deconstructive feelings like anger or anxiety were less prone to demonstrate this helpful educational manner when coping with difficulties.

The results of the study display that there is a meaningful negative relationship between negative emotions (anger and anxiety) and teachers’ resilience. It can be stated that anxiety, sadness, and worry have a deconstructive connection with resilience (Anyan and Hjemdal, 2016). A few scholars observed, for example, that deconstructive feelings consisting of stress and sadness had deconstructive influences on the growth of resilience (Galatzer-Levy et al., 2013; Yu et al., 2015). Likewise, negative feelings can be regarded as unfavorable sources that restrict the growth of resilience in humans. This means that humans subject to excessive degrees of risk elements, like deconstructive feelings, would possibly have low degrees of resilience. Moreover, it is logical to deduce, based on the challenge model of resilience that greater degrees of deconstructive feelings, namely, sadness, worry, and anxiety, bring about lower degrees of resilience.

Furthermore, those who encounter less deconstructive feelings are more capable of having pleasurable emotions and encountering fulfillment in their lives, according to the applied system of resilience. This will help them, in specific, with controlling their manners and their feelings. The moment individuals can control their feelings; they can build greater self-assurance and internal resources, like problem-solving, self-awareness, and self-efficiency, thereby enhancing their resilience growth (Liu and Ngai, 2019).

## Conclusion and Implications

Educators’ feelings have turned into a crucial emotional dimension of working to build educators’ professionals in the academic setting. This research has important practical suggestions for language educators, language learners, and teaching material developers. Educators in the context of language education can elevate inspiration and achievement by building constructive emotions and reducing deconstructive ones. Enhancing constructive emotions and considering destructive emotions will increase teachers’ resilience. It is advisable for language educators to acquire more psychological knowledge, become familiar with the main notions and elements of educators’ emotions, portray knowledge in their practice and temper, develop students’ perspectives of educators’ achievement in the L2 classes, and elevate their degree of resilience by focusing on the copying techniques. Since it should be precarious to mention deconstructive emotions too much, educators can practice deemphasizing the deconstructive emotions. This is because if educators portray such dangerous elements in the class, some learners will end up coming out badly in this regard. Research on educators’ emotions proposed that instruction is an emotional effort and that educators’ emotions are connected to their wellbeing and the excellence of their instructions (Brackett et al., 2010; Frenzel, 2014).

As stated by Fredrickson and Joiner (2002) from a theoretical point of view, the practice of constructive emotions can be regarded as a source that people can vigorously access and make use of. Helping educators become conscious of the existence and power of their enjoyment in the class can enhance their wellbeing and eventually turn them more resilient when encountering tension and worry. Indeed, educators are the main effective individuals who can build a constructive class atmosphere. Furthermore, their emotional support affects how learners perceive them. Educators’ educational success is primarily related to intellectual skills and scholastic and professional knowledge in the context of EFL. However, educators with both emotional and qualified literacy skills are most approved of by learners. Educators who have a constructive outlook toward classes and contents have more inclination, enjoyment, and excitement for work and class; thus, they can convey a positive mood to their learners throughout the course and successfully manage their lessons and they are more resilient during facing challenges in the journey of language teaching.

It is advisable to carry out several educator training programs that can encourage language educators for successful and up-to-date educational programs. Moreover, it is recommended to organize several relevant workshops to lessen educators’ anxiety, and these workshops will introduce and teach educators successful strategies for relieving serious stress and instructional anxiety. Language educators may also be motivated to read some impactful content to increase their self-esteem. This eventually reduces stress during the educational cycle. Likewise, the outcomes assist the teaching material developers in the sense that they can design assignments, activities, and reading materials that take into account the emotional condition of the educators so that they can cope with challenges without problems. This study has a psychological suggestion for syllabus designers so as not to avoid the part emotions play in syllabus design. Tasks and assignments that emphasize the role of educators’ feelings and how learners interpret educators’ passions and their influences on educators’ resilience need to be designed. As stated by Pekrun and Linnenbrink-Garcia (2014), a class is an emotional setting. Educators and learners develop a close and connected social connection that allows them to experience numerous emotions together by spending all their time together in this emotional setting. It is necessary to build educator resilience through a comprehensive educator training program in scholastic settings as educators are at the forefront of the fight against challenges, where emotional conditions and preparation are changing instructional results around the world (Derakhshan et al., 2020).

With all the time spent together in an emotive setting, educators and learners can develop social connections that bring them closer to one another and connect them to encounter feelings together. Therefore, it can be stated that scholastic situations exist with some kinds of emotions such as enjoyment, optimism, anger, burnout, and anxiety (Pekrun and Linnenbrink-Garcia, 2014). Further studies aimed at investigating the relationship between educators’ feelings and other notions like management skills are stimulated by the presence of TES. For instance, it would be interesting to find out how documents on educators’ enjoyment, anger, and anxiety are connected to their endeavors to be involved in the educational cycle. It is hoped that the presence of the TES stimulates further studies focused on examining the relations between educators’ feelings and their concepts of PP. The results of the study emphasize the outlook that emotions are at the core of language learning and since the role of positive emotions is assured, it can be a bright prospect for the development and growth of PP.

## Data Availability Statement

The original contributions presented in the study are included in the article/supplementary material; further inquiries can be directed to the corresponding author.

## Ethics Statement

The studies involving human participants were reviewed and approved by the China University of Geosciences (Wuhan) Academic Ethics Committee. The patients/participants provided their written informed consent to participate in this study.

## Author Contributions

All authors listed have made a substantial, direct, and intellectual contribution to the work and approved it for publication.

## Conflict of Interest

The authors declare that the research was conducted in the absence of any commercial or financial relationships that could be construed as a potential conflict of interest.

## Publisher’s Note

All claims expressed in this article are solely those of the authors and do not necessarily represent those of their affiliated organizations, or those of the publisher, the editors and the reviewers. Any product that may be evaluated in this article, or claim that may be made by its manufacturer, is not guaranteed or endorsed by the publisher.
